# Advancements and Applications in the Composites of Silk Fibroin and Graphene-Based Materials

**DOI:** 10.3390/polym14153110

**Published:** 2022-07-30

**Authors:** Zhimin Xu, Yujie Ma, Huanyan Dai, Shuang Tan, Bing Han

**Affiliations:** Department of Oral and Maxillofacial Surgery, School and Hospital of Stomatology, Jilin University, Changchun 130021, China; xuzhimin@jlu.edu.cn (Z.X.); mayj20@mails.jlu.edu.cn (Y.M.); daihy20@mails.jlu.edu.cn (H.D.); tanshuang20@mails.jlu.edu.cn (S.T.)

**Keywords:** silk fibroin, graphene, polymers, Cocoon, hybrid composites, biocompatibility

## Abstract

Silk fibroin and three kinds of graphene-based materials (graphene, graphene oxide, and reduced graphene oxide) have been widely investigated in biomedical fields. Recently, the hybrid composites of silk fibroin and graphene-based materials have attracted much attention owing to their combined advantages, i.e., presenting outstanding biocompatibility, mechanical properties, and excellent electrical conductivity. However, maintaining bio-toxicity and biodegradability at a proper level remains a challenge for other applications. This report describes the first attempt to summarize the hybrid composites’ preparation methods, properties, and applications to the best of our knowledge. We strongly believe that this review will open new doors for coming researchers.

## 1. Introduction

Silk fibroin (SF), a natural polymer fibrous protein extracted from silk, has been explored in many fields, such as tissue engineering, regenerative medicine, and drug delivery [1,2,3,4], owing to its excellent characteristics, for example, the ease of preparation and modification, outstanding biocompatibility and biodegradability, stable mechanical properties, and low immunogenicity [5,6,7]. However, further studies on enhancing its mechanical properties and controlling the degradation rate without compromising its inherent properties must be carried out for more extensive use across fields [8].

**Figure 1:**
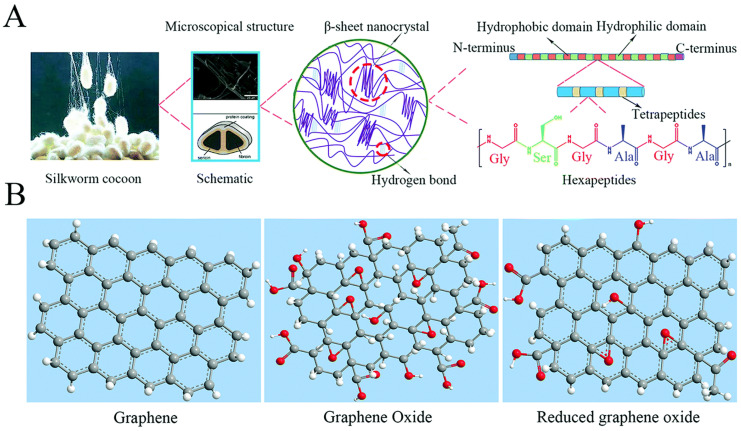
The structures of SF and graphene-based nanomaterial building blocks: (**A**) the hierarchical structure of SF; (**B**) the structures of GBNs. Reproduced with permission from [15].

Graphene and its derivatives (GDs) include few-layered graphene (FLG), ultrathin graphite, graphene oxide (GO), reduced graphene oxide (RGO), and graphene nanosheets [9]. As a kind of widely used two-dimensional nanomaterial, GDs have exhibited much potential in the bone regeneration fields due to their outstanding mechanical properties, electrical conductivity, and atomic structural stability [10,11]. What has been concluded is that GDs can be applied in the conduction of bone repair scaffolds and the development of osteogenic cell activity and bone formation, relying on sufficient mechanical enhancement and desired electrical stimulation, respectively [12,13]. However, GDs also present some shortcomings, including a slow biodegradation rate, dose-dependent biotoxicity, and so on [14]. Therefore, the combination of GDs and other biomaterials should be investigated further to improve their physicochemical properties in biomedical fields. The structures of SF and graphene-based nanomaterial building blocks has been shown in the Figure 1.

**Figure 2:**
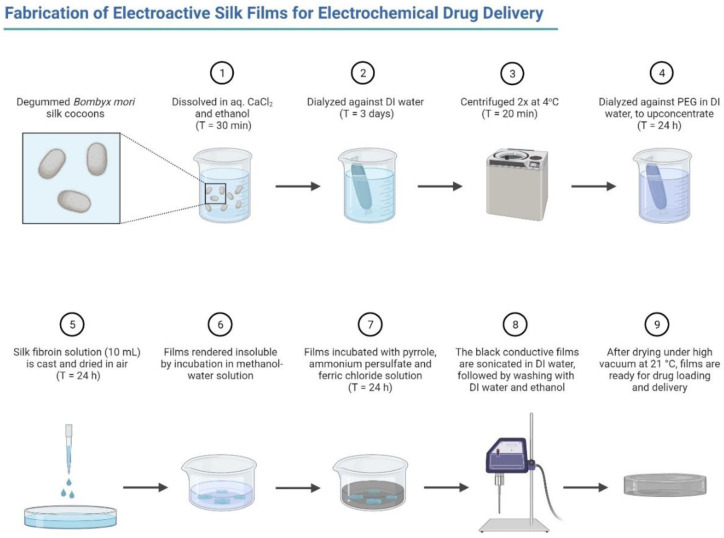
Fabrication of electroactive silk forms for electrochemical drug delivery (generated by Biorender).

**Figure 3:**
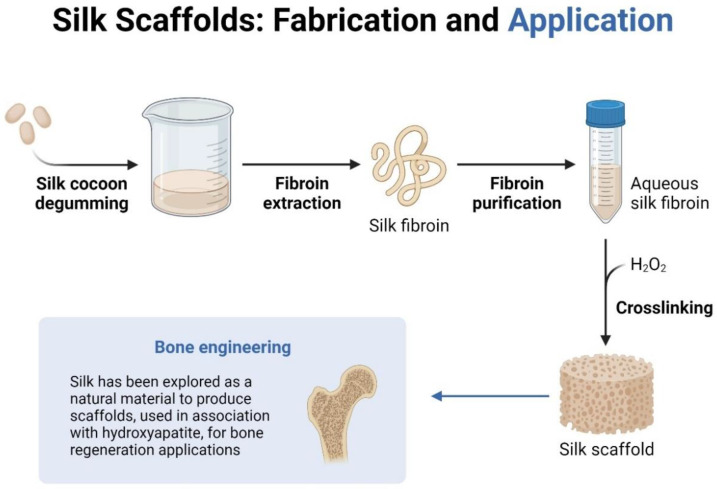
Silk scaffolds, fabrications, and applications in bone engineering (image created in Biorender).

Recently, the combined application of SF and graphene-based materials has come to be regarded as a popular topic in biomedical fields such as tissue engineering, drug delivery, and some dental fields as shown in Figure 2 and Figure 3. This review highlights the properties of SF and graphene family materials, the applications combining these two kinds of materials, and lists existing challenges and future perspectives.

## 2. Preparation Methods for SF and Graphene-Family Materials

Graphene and graphene-based materials, as novel and promising biomaterials, have been applied in bone tissue engineering by incorporating them into various scaffolds, such as silk-based scaffolds. The combination of SF and graphene-family materials can counteract the deficiencies of each to broaden their applications in biomedical fields. SF and graphene-based derivates can be combined by either physical (non-covalent) binding or chemical (covalent) binding, or both [16]. The main physical binding methods include layer-by-layer (LbL) assembly and treating the mixture of SF and graphene-family materials with freeze-drying or air-drying [17,18]. The chemical binding methods include chemical crosslinking, photochemical crosslinking, and electrochemical microstamping [19,20]. In the case of GO, rGO and SF can easily be modified, and covalent and non-covalent bonds may be generated simultaneously during the modification processes of SF, GO, and rGO, such as via crosslinking methods, in turn, modulating the pH before homogeneous mixing [21,22].

### 2.1. Freezing-Drying

Freeze-drying, a gentle dehydration method, is commonly used to dry porous scaffolds by sublimating the ice particles formed in the freezing process [23]. The size of ice particles in the freezing process is closely related to the growing velocity of ice crystals, growing time, growing history, and other aspects [24]. However, with the increment of the GO content, the increasing viscosity of the blended solution caused by the formation of the hydrogen bonding interaction between SF molecules and GO nanosheets resists the formation of larger ice crystals, thus leading to a decrease in the scaffold’s pore diameter. What is noteworthy is that the porosity of the hybrid scaffolds is 92–94% despite the variables not causing damage to the migration or proliferation of cells or vascularization [23].

### 2.2. Layer-by-Layer (LbL)

The LbL assembly technique may also be employed to fabricate a kind of robust microcapsule containing silk fibroin and graphene oxide with controlled permeability [22]. This method relies on the electrostatic attraction formed by the deposition of bilayers, consisting of the SF layer modified by poly-(L-lysine) with positive charges and the negative GO layer on the original silica spheres with negative charges.

Nanomembranes with excellent uniformity and overall integrity, containing alternating layers of SF and GO, were prepared using the spin-assisted layer-by-layer (SA-LbL) technique [18]. To do so, 5 nm-thick SF layers were covered with 0.95 nm-thick GO sheets with 69% coverage density. Increasing the number of GO layers could regulate the increase in the GO volume concentrations and the total SF/GO membrane thickness. Yarn-on-yarn abrasion has been discussed in detail [25,26].

### 2.3. Electrospinning

Electrospinning has become a standard and notable method to fabricate scaffolds recently. It has been demonstrated that scaffolds prepared by electrospinning can simulate the nanofiber bundle components of the bone ECM and wound healing [27,28,29,30]. Taking the form of mats with parallel or random fibers, the scaffolds make it challenging to process or shape the nanofibers, due to insufficient mechanical properties, without the help of particular methods such as patterned electrospinning [31,32]. The scaffolds with parallel nanofibers possess better tensile properties than those with randomly oriented nanofibers [33,34,35]. The reduced GO and SF were blended and fabricated into nanofibers by electrospinning, which indicated that the presence of reduced GO could increase the thermal stability, mechanical properties, cell viability, and osteogenic differentiation in low-concentration RGO composites [36].

### 2.4. Vacuum-Assisted Assembly

Vacuum-assisted assembly has been referred to as a widely used method for fabricating graphene-based flexible “paper” materials [37,38,39,40], which have been regarded as promising carbon-only replacements for inorganic-based nanocomposites with outstanding mechanical properties. To overcome the limited possibilities for generating electrical conductivities and long-term stability under wet conditions [41,42,43], an approach of strengthening GO papers using the chemical crosslinking method has attracted much attention [44].

### 2.5. Photochemical Crosslinking Method

A photochemical crosslinking method has been employed to fabricate tough nanocomposite hydrogel consisting of regenerated silk fibroin (RSF) and graphene oxide [20]. The formation of dityrosine crosslinks was derived from tyrosine amino acid residues in RSF, relying on the ruthenium-mediated photochemical crosslinking technique [45]. During the initial 2-min exposure to white light, the RSF hydrogel exhibited a non-adhesive state. In contrast, the RSF/GO composite solution formed an adhesive hydrogel, suggesting a GO-induced adhesive nature the RSF/GO composite hydrogel. After an additional 8-min light exposure, a non-adhesive hydrogel RSF/GO composite hydrogel was produced. In addition, methanol treatment has been widely used in the composite fabrication process [18,20]. Methanol treatment was demonstrated to generate less ordered RSF secondary structures, along with the formation of more voids, transforming randomly coiled fibroins into β-sheets, thus increasing the silk II content [20]. It has previously been reported that such less-ordered secondary structures form rapidly, relying on methanol induction between the crystalline and amorphous domains in RSF cast films, which enables higher water uptake [46]. The Young’s modulus of β-sheet structures was 22 GPa [47]. Methanol treatment may weaken the Young’s Modulus of SF films, related to the 45% fraction of β-sheet crystals, in intensely methanol-treated silk fibroin [48]. Therefore, after methanol treatment, pristine SF membranes presented a slightly higher Young’s modulus (10 GPa) than LbL SF membranes, probably due to the extensive use of methanol treatment [18].

### 2.6. Surface and Structural Properties

Parts of the areas making up the deposited graphene on the SF/GO conductive fibrous scaffolds may appear darker, indicating some small fragments of graphene being stacked [49]. Dispersion conditions without stacking graphene in the SF matrix can determine the composite performance to a large extent. Therefore, surface uniformity should be guaranteed as the first step. The fiber diameter of the G/SF scaffolds was more significant than scaffolds without added graphene, and the viscosity enhancement was regarded as the dominant factor in increasing the diameter variation. However, when the content of graphene reached 4 mg mL^−1^, the diameter was suddenly decreased by enhanced conductivity produced in the process of electrospinning, increasing the elongational forces. Too-high graphene contents hindered the complete dispersion of graphene flakes in the solution, negatively impacting the fiber diameter nonuniformity.

To investigate the effects of graphene on the physicochemical properties and in vivo cellular responses of silk fibroin scaffolds, Ding et al. fabricated porous 3D SF/graphene scaffolds with different contents of graphene (0, 0.5, and 2%) [50]. It was observed that the graphene nanosheets were homogeneously dispersed in the SF scaffolds. Moreover, what was interesting was that with a higher graphene content, the scaffolds exhibited decreased pore diameters [23,50]. This may have been related to the increased viscosity of G/SF scaffolds, which made it challenging to move water molecules freely and resulted in the limited formation of larger ice particles [50]. Therefore, moderate graphene contents in the SF scaffolds presented a synergistic effect on cell proliferation, while excess graphene was unfavorable for stem cells [23,50].

The uniformity of topography may depend on the ratio of SF to GO. In pure configurations, GO has the appearance of a slightly cracked sheet with grooves in the configuration. Meanwhile, silk fibroin appears to have a largely smooth surface, according to previous studies [51]. As observed in the previous studies of SF/GO composite films [52], surface irregularities increased gradually with the silk fibroin content in different SF/GO mixtures.

The spin-assisted layer-by-layer (SA-LbL) technique was employed and assembled a type of ultra-robust nanocomposite membrane containing GO sheets and silk fibroin [18]. Silk fibroin layers covered with GO sheets presented a uniform topography with a root-mean-square (RMS) roughness of 4.3 ± 1.9 nm and a 69 ± 9% graphene oxide surface coverage density. The average bilayer thickness of a typical LbL SF/GO membrane was about 5.4 nm, constituted by 5 nm-thick SF layers and 0.95 nm-thick GO flakes overlapping each other. A lack of evident aggregation and self-folding is beneficial for maximizing the interfacial interactions between silk domains and inhomogeneous matrix surfaces [53]. The dense network of weak interactions between modestly aggregated silk fibroin and graphene oxide flakes in around a 5 nm-thick biolayer could enhance the reinforcing effect by forming challenging molecular interphase zones of confined silk material [18].

**Figure 4:**
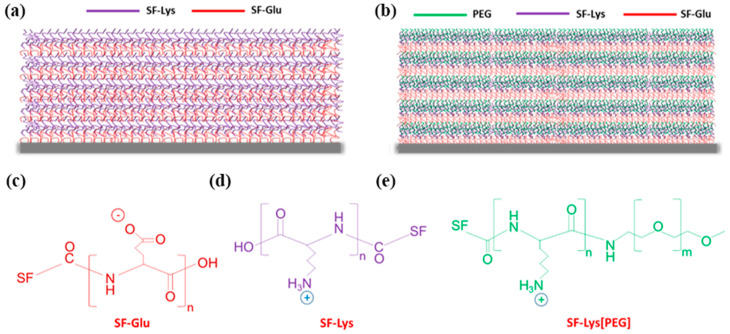
LbL silk ionomer films fabricated in this study: (**a**) ((SF)-poly-l-glutamic acid (Glu)/SF-poly-l-lysine (Lys))_5_ and (**b**) (SF-Glu/SF-Lys[poly(ethylene glycol)(PEG)])_5_; chemical structure of silk ionomers: (**c**) silk fibroin (SF)-poly-l-glutamic acid (Glu), (**d**) SF-poly-l-lysine (Lys), and (**e**) SF-Lys[poly(ethylene glycol) reproduced with permission from [64].

The robust LbL microcapsules containing poly-amino acid-modified silk fibroin reinforced with graphene oxide controlled permeability [22]. The planar GO flakes had a capacity of homogeneous dispersion and a sonication-controlled microscale lateral size (<500 nm after sonication). The GO sheets showed a thickness of 1.0 ± 0.1 nm and predominantly single/double flakes, according to previous reports [54,55,56]. After the dissolution of the silica core, the microcapsules with a diameter of about 3.7 μm seemed robust enough to preserve the spherical geometry. The hollow shells possessed a uniform morphology with large and small wrinkles associated with randomly folded flexible GO nanosheets. The hybrid (SF-PL/GO)_n_ shells, with an increasing thickness from 5 ± 0.5 nm to 23 ± 2 nm, along with an increasing number of bilayers, exhibited linear growth, indicating the absorption of the silk ionomer/GO composite only on the top of the outermost layers, and a restricted interlayer diffusion [57,58], mainly due to the limitation of the impermeability of GO nanosheets [22]. Significant changes have been revealed in the roughness of the hybrid shells, which could be summarized by the fact that with increasing numbers of bilayers, the shell microroughness increased from 3.8 to 8.4 nm, accompanied by a change from a relatively smooth-surface topography to a rough and aggregated one, mainly caused by the increment of randomly oriented wrinkles. Investigating the LbL hybrid (SF-PL/GO)_3.5_ shell permeability at different pH conditions was the key point of this research. With the increase in pH value from 2.0 to 11.5, the molecular weight of the dextrans that were permeable for the hybrid shells revealed a descending trend with the change for almost all, except 2000 to 250 kDa. In addition, the porosity of the SF/GO shells controlled by pH was much lower compared to pure silk shells. Nevertheless, previously reported robust and pH-responsive thin-shell LbL silk-based microcapsules demonstrated reversible variations in shell permeability, where the permeability was significantly enhanced in acidic and basic conditions, along with pore sizes becoming much larger, thereby accelerating the permeability of large macromolecules irrespective of environmental conditions [59]. The transformations of pH-dependent permeability for (SF-PL/GO)_3.5_ hybrid microcapsules can be ascribed to the transition from extended molecular chains to random coils on account of reduced charge density along with increasing pH values [60,61,62], thus resulting in the shrinkage of SF-PL chains with gradual compaction of the shells and a decrease in shell porosity. A phenomenon of the absence of significant swelling behavior for (SF-PL/GO)_3.5_ composites in highly acidic (pH 2.0) and primary (pH 11.5) conditions was inconsistent with the great-volume swelling (up to 800%) of homogeneous silk ionomer capsules previously reported [59]. This was noteworthy and could be attributed to the interface formed by the tightly overlapped, folded GO nanosheets wrapping with silk chains, of which ionic paring and hydrogen bonding between GO sheets and silk chains were the main reasons to maintain the stability of composite shells in severe pH conditions [63], as shown in Figure 4.

The strong SF/GO composite hydrogel films with layered structures, forming a loose three-dimensional (3D) network and mimicking the structure of nacre, relying on the force balance between electrostatic repulsion and hydrogen bonding interaction, possessed an SF uniformly coating on GO sheets, in which a single layer had lateral dimensions of several micrometers and a thickness of about 0.8 nm [21].

Due to the GO sheets, a corrugated morphology was formed on the PLGA/TSF/GO nanofiber surfaces, resulting in micropores or mesopores [65]. TSF played a crucial role in accelerating the nucleation and growth of HA on PLGA/GO scaffolds exposed to simulated body fluid. A strong interaction was formed between the mineral deposits and nanofibers. Mineral particles, consisting of three-dimensional arrays of acicular HA crystals, exhibited a flocculent morphology and needle-like shape, with 40−50 nm lengths and 2–5 nm, further transforming into the micropores or mesopores.

The porous SF/GO composite scaffolds yielded by a green fabrication method presented a continuous pore structure and a remarkable 92–94% porosity. The morphology and pore diameter partly depended on the GO content [23]. After GO from 0 to 1 wt%, the pore structure transformed from leaf-shaped or spindle-shaped into elliptical-shaped, with a decrease in the average pore diameter from 102 to 81 μm and an enhancement in the uniformity of the pore size. The decrease in the average pore diameter was relevant primarily to the increased viscosity of the blend solution caused by the formation of hydrogen bonding between SF molecular and GO nanosheets, resulting in the complex and slow movement of the water molecule as a barrier to the formation of ice particles. The XRD results indicated a typical silk II structure mixed with a little silk I structure, and uniform dispersion of GO in the SF matrix without aggregation in hybrid SF/GO scaffolds.

### 2.7. Mechanical Properties

It has been demonstrated that adding graphene can enhance the mechanical features of composite materials [66,67]. The intermolecular forces formed between SF and graphene can impede the movement of the polymer chains, which can explain graphene’s function by enhancing Young’s modulus and the tensile strength of composites [49,68]. Another reason is the increase in crystallinity degree, which can densify the crosslinking points of amorphous molecules and then control them, to prevent them from slipping off in the mechanical test process [69]. However, an excessive graphene content may harm the mechanical properties. Yang et al. found that Young’s modulus and the tensile strength of G/SF composite scaffolds with a graphene content reaching 4% were lower than those of the pure SF scaffolds [49]. The fact might be attributed to the van der Waals force of the graphene nanosheets, which led to restacking when adding graphene to the SF solution [49]. Zhao et al. put forward a theory that a critical point existed for the graphene content that affected the mechanical properties [67]. A content lower than this critical point could lead to the total dispersion of the exfoliated graphene nanosheets in the SF solution. On the contrary, a higher content than the point could facilitate nanosheets’ stacking together. If this is the case, then a suitable graphene content is vital to the mechanical properties of G/SF composite scaffolds.

Among the graphene-based materials, except for the graphene, graphene oxide and reduced graphene oxide also contributed a lot to enhancing the silk-matrix biomaterials’ mechanical performance. Huang et al. [21] reported the preparation of SF-GO solid composite films through the transition from stable SF-GO hybrid hydrogel via a solution-casting method, which is inexpensive, simple to operate, time-saving, and easy to scale up. A composite film containing 15 wt% SF exhibited a high tensile strength of 221 ± 16 MPa, a failure strain of 1.8 ± 0.4%, and a high modulus of 17.2 ± 1.9 GPa. The excellent mechanical features can be attributed to its high GO content (85 wt%), dense, layered structure, and the strong hydrogen bonding interaction between SF chains and the GO sheets.

An SF-GO layered film was fabricated through another method, vacuum-assisted filtration, and showed excellent mechanical properties [19]. A sandwich-like structure was established by putting the film between two pieces of thin aluminum foils to induce an electrochemical reduction. GO was reduced by Al ions and the resulting SF-rGO films got improved mechanical properties, such as a tensile strength from 150 to 300 MPa and a modulus from 13 to 26 GPa, though the breaking strain of 2.8% decreased to 1.5%.

In contrast to the above two SF-GO composites films [19,21], the ultra-robust GO-SF nanocomposite membranes have presented superb mechanical performances [18]. The rising Young’s moduli of the SF/GO nanocomposites were demonstrated to be linearly related to the graphene oxide concentration; the highest value was 145 ± 4 GPa at 23.5 vol% of graphene oxide, much higher than the modulus values of the above two SF/GO composite films. The interphase reinforcement mechanism in nanocomposites suggested that extended interphase zones formed between the two constituents were primarily related to the enhanced intermediate properties [70,71,72,73], leading to the doubled volume concentration of the effective high-modulus filler [18]. Gradual delamination of the LbL membranes, resulting from the local wrinkling of graphene oxide flakes in bucking experiments, was likely to have caused the consistently higher tensile modulus than the compressive modulus [41,74,75]. With the ultimate stress reaching over 300 MPa and the ultimate strain within 1.0 ± 0.4%, the SF-GO methanol-treated nanocomposite also exhibited superior toughness, as high as 2.2–2.4 MJ m^−3^, with the highest toughness of 3.4 MJ m^−3^ recorded for some samples.

Except in the form of films, the robust (SF-PL/GO)_n_ hybrid microcapsules were fabricated and exhibited remarkable stability at high concentrations of dextran solution, without noticeable buckling, over a wide range of dextran concentrations from 0 to 25%. However, the microcapsules fabricated by only silk ionomers, without graphene oxide sheets, presented partial deformation of silk-matrix capsules at the 4% dextran concentration. They even led to a collapse when the dextran concentration reached 12% [22]. The chemical crosslinking method can enhance the mechanical properties of biomaterial [76], and a comparison of mechanical properties between crosslinked pure silk ionomer microcapsules and non-crosslinked silk-graphene oxide composite shells indicated that graphene oxide acted as a primary reinforcing factor of the solid mechanical capacities of (SF-PL/GO)_n_ shells. The elastic modulus of reinforced (SF-PL/GO)_n_ shells exhibited a very high value, around 470 MPa, surpassing typical values for pure polymers in the swollen state or LbL protein shells, indicating the remarkable effect of graphene oxide on the mechanical properties of shells [77,78].

To some extent, the standard configuration of the scaffolds for tissue engineering could affect the mechanical properties of the composite biomaterials. Biomineralized poly(L-lactic-co-glycolic acid) (PLGA)/graphene oxide/tussah silk fibroin (TSF) nanofiber scaffolds with multiple orthogonal layers were fabricated through the electrospinning method [65]. Multiple orthogonal layers endowed scaffolds with a higher compressive modulus and stress than randomly oriented allayed nanofibers. The incorporation of 1 wt% GO decreased the fiber diameter of nanofibers compared to scaffolds without GO, probably derived from the increase in the conductivity of the electrospinning solution. Moreover, adding GO also resulted in a higher compressive modulus and stress.

The Young’s modulus of the tough RSF/GO nanocomposite hydrogels reached as high as ∼8 MPa, and the tensile toughness was as high as ∼2.4 MJ/m^3^ [20]. The RSF/GO hydrogels under the dehydrated state possessed a greater toughness and ultimate tensile strength than those in the swollen equilibrium state. It was demonstrated that methanol post-treatment endowed hydrogels with a better Young’s modulus. After methanol treatment, the dehydrated RSF/GO hydrogels exhibited the highest Young’s modulus of 22 MPa among all samples. However, NaBH_4_ treatment could significantly weaken the mechanical properties of the RSF/GO nanocomposites, which could probably be ascribed to the low crosslink density and the disruption of a disulfide bond in RSF.

The compressive moduli of a kind of SF/GO hybrid scaffold incorporated with 0.1, 0.2, 0.5, and 1.0 wt% GO fabricated via a freeze-drying green method were 19.14, 19.33, 21.73, and 24.32 kPa, respectively, higher than that of the new SF scaffold (14.02 kPa) [23]. The uniform dispersion of GO in the SF matrix, and the intermolecular forces between SF and GO nanosheets, improved the mechanical properties.

Most SF/GO composite materials presented an improved mechanical performance compared to their counterparts. The hybrid materials were typically stiffer than pristine SF materials and tougher than GO materials [79]. The excellent mechanical properties primarily relied on the potent combination of SF and GO at the macroscale, which can be ascribed to the strong interactions at the interfaces of the SF/GO nanocomposites, including polar-polar hydrogen bonding and hydrophobic-hydrophobic interactions [80,81,82] Acting as the driving forces to facilitate the transitions of SF from random coils or helixes into β-sheet crystals, these interactions at the chemical bonding and molecular scales contribute greatly to the interlocking of fibroin chains and further lead to the enhancement of the toughness and strength of composite materials [83].

### 2.8. Thermodynamic and Electrochemical Properties

Thermodynamic properties of G/SF conductive scaffolds were investigated by differential scanning calorimetry (DSC) to explore the effect of graphene on the crystallization of the scaffolds [49]. Results showed that the stable reticulation flat structure in the fiber of the fibrous scaffolds might be improved by graphene, resulting in an increase in the glass transition temperature. In addition, the melting temperature also increased gradually with the addition of graphene. Which formed a hydrogen bond between graphene and SF to fix the polymer molecular chain of the composite. As for the crystallinity level of SF molecules, G/SF scaffolds exhibited better effects than pure SF scaffolds.

Electroactive biomaterials can exert electrical stimulation on the cells growing on them. Electrochemical tests indicated that higher graphene contents endowed the G/SF scaffolds with higher conductivity and could carry electrical stimulation further to the cells [49].

It was demonstrated that the biocompatibility of SF and GO could make them a promising biomaterial in biological scaffold and tissue engineering. The isoelectric point (pI) of SF is 3.8–3.9 [84], which is lower than the pH of GO solution (4.6), resulting in an irregular precipitate after mixing SF and GO solutions. The main reason is that the weak electrostatic repulsion force between GO sheets at pH = 4.6 cannot counteract the attraction force caused by the hydrogen bonding between SF chains and GO sheets [21]. A certain amount of ammonia solution is added to the GO solution to modulate its pH value to 10, to increase the negative charges of SF chains and GO sheets.

### 2.9. Hydrophilic, Hydrophobic, and Water Soluble

A hydrophilic surface of a biomaterial possesses a slight contact angle. Some studies have concluded that moderately hydrophilic surfaces (water contact angle between 20° and 55°) are beneficial to cell attachment and growth on the scaffolds [85,86,87,88]. However, the composites with increasing graphene contents showed increasing contact angles, revealing hydrophilicity with a gradually decreasing trend versus the pure SF scaffolds. SF contains various hydrophilic groups, such as amine, hydroxyl, and carboxyl [89]. Nevertheless, the graphene layer is hydrophobic [90,91]. For the electrospun G/SF scaffolds with some contents of graphene (1–4%), the contact angles were within the suitable range for cell growth [49].

Water stability plays a vital role in the different applications of in vitro and in vivo scaffolds. The porous SF/GO hybrid scaffolds fabricated by a green method exhibited excellent water stability by incorporating 30 wt% and 40% glycerol into scaffolds, mainly due to the improved formation of the stable silk I and silk II crystal structures induced by glycerol, which was used as a plasticizer [23].

### 2.10. Cytocompatibility and Cell Viability

Confocal microscopy analysis is usually adopted to reveal the morphological features of cells, and a CCK-8 assay is used to determine the viability and proliferation of cells in cytocompatibility tests. The results for SF/G composite scaffolds showed that rBMSCs displayed a spindle shape for all groups of scaffolds, meaning that the cells were widely spread on the scaffolds with distinct spread actin filaments [49]. As for the toxicity of scaffolds, the OD value of the CCK-8 assay indicated that rBMSCs could attach and proliferate normally on scaffolds, proving nontoxicity [49]. It is worth noting that the viabilities and proliferation of rBMSCs on G/SF scaffolds were affected by the graphene content; 4% graphene adversely impacted the cellular responses, and no significant differences could be distinguished between 1, 2, and 3% G/SF scaffolds and the pure SF scaffolds (*p* > 0.05). It can be concluded that the incorporation of graphene can transform the SF scaffolds into electronic conductors without compromising the inherent superb cytocompatibility of the SF matrix [49].

Regarding the cell behavior of rBMSCs on the porous G/SF scaffolds [50], 0 and 0.5% G/SF presented the best cell proliferation, and 0.5% G/SF displayed excellent osteogenic properties versus the other groups. Mineralization with HA, or increasing the specific surface area and pore volume, could promote cell adhesion, proliferation, and migration and further facilitate mass transport of nutrients and metabolites [65]. Tussah silk itself can render the surface hydrophilic, and mineralization can further improve hydrophilicity. This double function makes the composite scaffold a promising material in the tissue engineering field. Cell viability tests of the hybrid SF/GO scaffolds indicated a uniform dispersion and close adherence of MC3T3-E1 cells. The incorporation of 1 wt% GO presented a relatively poor function in terms of cell proliferation compared to the GO contents of 0.1, 0.2, and 0.5 wt%, which confirmed that it was only the moderate content of GO (rather than without GO or with excess GO) in the SF-based biomaterials that had a synergistic effect on the proliferation of MC3T3-E1 cells [23].

Graphene oxide (GO) possesses abundant functional groups and a large surface area, which can provide reactive sites for functionalization [92]. Previous studies indicated that peculiar functional groups of GO are favorable for regulating cell behaviors [93,94,95]. Attempts have been made to fabricate GO and SF composites, but the resultant materials did not resemble nanofibrous ECM [18,19]. Therefore, combining SF with GO into nanoscale composites remains an issue. In one study, SF nanoparticles were mixed with GO nanosheets to trigger the assembly of SF nanoparticles into oriented nanofibrils under the guidance of GO nanosheets, leading to the formation of SF/GO films with an enhanced modulus [42]. The redox reaction between SF and GO leads to the transition of SF molecules from random coils to β-sheets and the reduction of the oxidative groups in GO, resulting in the formation of SF nanofibrils, which become much denser as the GO content increases. It was verified that the SF films combined with 10% GO could accelerate early cell adhesion and induce osteogenic differentiation of hMSCs.

### 2.11. Biodegradation

When exposed to an enzyme solution, the scaffolds containing only SF or hybrid SF/GO exhibited a fracture and collapse phenomenon after 14 days of degradation and further corroded drastically at 28 days [23]. However, an increase in the GO content could endow the scaffolds with an enhanced capacity to resist enzyme degradation, thereby postponing the degradation process, and as such, maintaining a porous structure and more integral form for a longer time. The residual material of the SF/1 wt% GO composite scaffolds was 50.64%, much higher than that of pure SF scaffolds. It was demonstrated that the combination of hydrogen bonding or some other intermolecular forces formed between amphipathic GO nanosheets and the silk backbones with alternating hydrophilic and hydrophobic nanoscale domains reduced the diffusion of the enzymatic solution into the scaffolds and subsequently led to a lower degradation rate [23].

Lyu et al. [43] reported the construction of layered ZnO nanotubes/silk fibroin/graphene oxide nanostructures (SF/GO/ZnO) on pure zinc substrates via anodizing and SF/GO self-assembly. The osteogenic drug dexamethasone (Dex) was adopted as a coating layer to improve the osteogenic potential of the composites. The results demonstrated that it might be the unique structure of GO and the active oxygen generated during the degradation of ZnO that endowed excellent bactericidal and osteogenic activity to the composites. The cell membrane of bacteria could be destroyed by the sharp part of the GO nanosheet structure through direct mechanical contact with bacteria. The cells presented a stretched-in-different-directions condition on the composite with SF/GO coating. In contrast, the cells on the pure zinc or anodized zinc groups were elongated, proving that an SF/GO coating positively promoted cell proliferation. The fact that the composition of SF is similar to that of collagen, the main component of the extracellular matrix, was beneficial to the cellular behaviors. Meanwhile, GO had a great adsorption ability in terms of serum protein, causing a higher density of adhesion molecules that could react positively for cell attachment and growth.

### 2.12. SF Modification

Silk fibroin (SF) is a natural protein polymer and promising biomaterial. Modifications of SF have attracted growing interest in expanding SF applications. For modification of silk fibroin, some reactive reagents such as isocyanate, cyanuric chloride, and acid anhydrides have been used. Chemical modification of silk fibroin was carried out through serine amino acid residues, which showed that surface modification on native SF through serine residues was practicable and had the advantage of an increased β-sheet structure [96]. Isocyanates are a family of highly reactive chemicals, which are reactive toward not only amino groups but also carboxyl and alcohol/phenolic hydroxyl groups, meaning 3.5 mol% of residues in silk fibroin could participate in modification using 2-methacryloyloxyethyl isocyanate [97]. Besides the isocyanate, cyanuric chloride is also an effective coupling agent for the attachment of functional molecules to SF [98].

However, most modification agents have lower reactivity than isocyanate, cyanuric chloride, and anhydrides toward the reaction site of amino acid side-chains in SF protein. The most extensively utilized modification technique is the derivatization of the carboxylic acid residues via carbodiimide coupling with primary amines [99,100]. To expand on the functionalization strategies for SF, a group of researchers developed a modification method. They used diazonium coupling chemistry, which functionalized the tyrosine residues in SF to control the structure and hydrophilicity of SF protein [101]. Similarly, modification of silk protein by gene engineering was reviewed [102].

## 3. Applications in Biomedicine

A 3D printable electroconductive biocomposite bioink was fabricated based on glycidyl methacrylate silk fibroin (SB) and reduced GO, which possessed electrical conductivity, thermally stability, and the potential to become a promising biomaterial for neural tissue engineering [103].

A conductive SF/G fibrous scaffold with 3% graphene was developed via an electrospinning technique, possessing excellent electrical conductivity and extraordinary physicochemical features, which made it a promising biomaterial for potential applications in local electric fields or local ionic currents for cell cultures, biological interfaces, or animal studies [49].

**Figure 5:**
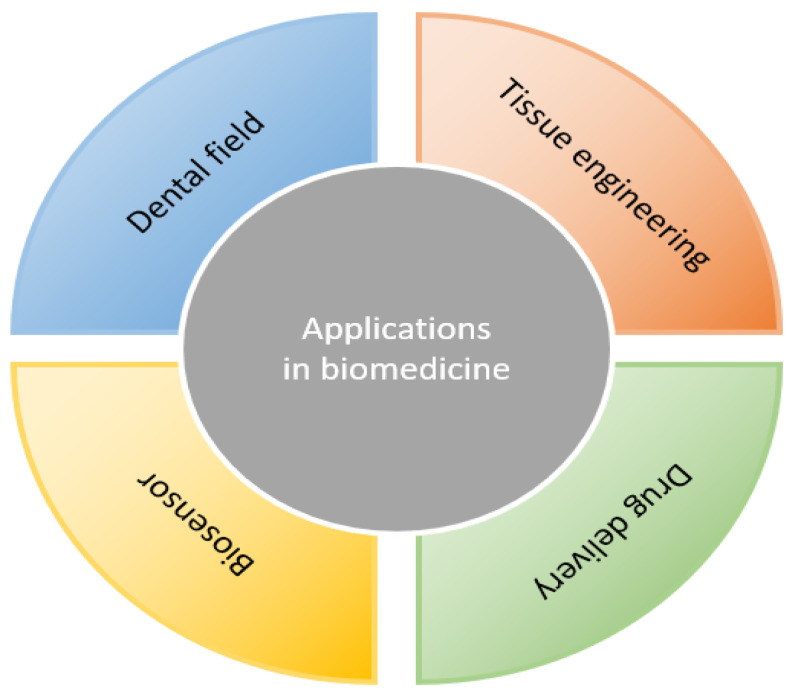
Applications of the combination of SF and graphene-based materials in biomedicine.

It is widely believed that SF is a biocompatible material useful for preparing cellular scaffolds for tissue engineering. A composite of the two materials has already been proposed for several biomedical applications [18,104]. Various SF matrix materials can be mixed with graphene-based materials in biomedical applications to improve their mechanical properties, electrical and thermal conductivity, and microstructure. Meanwhile, graphene-family biomaterials can also be modified using SF for better biodegradation and biocompatibility. The combination of these two materials may offset their drawbacks, making the composites a promising biomaterial for applications in biomedical fields (Figure 5).

### 3.1. Applications in the Dental Field

SF and graphene-family materials have been widely used in the biomedicine field of tissue repair and regeneration. The composites containing both are considered to exert better effects on tissue engineering. Although graphene-family materials have been widely introduced into the dental field, the use of the SF/graphene family can provide a more effective method [105]. In the dental regeneration field, a scaffold is often required for stem cell-based therapy to deliver cells and growth factors to the injured sites [51]. The SF/GO composite membranes produced by Rodríguez-Lozano exhibit excellent biocompatibility and significantly promote the cell proliferation of periodontal ligament stem cells (PDLSCs) compared to pure SF or pure GO groups, except for greatly enhancing the mechanical properties of the SF-matrix [51]. Recently, Vera-Sánchez et al. developed SF/GO and SF/rGO composite films via LbL deposition. The results showed that the cellular responses of the rGO group were more significant than those of the GO group at the same concentration [52]. In other words, within a specific concentration range, the cell proliferation rate improved due to the increment of the reduction degree of the graphene-family materials. In addition, the group investigated the best configuration (rGO:rSF at a 1:3 ratio) for cell proliferation, and differentiation and gene expression analysis showed that SF-graphene composites could induce the differentiation from human PDLSCs into cementoblast without any growth factors. These findings implied that the composites have great potential in regenerative dentistry and treating oral diseases, including common periodontal diseases.

A compelling SF/GO film can achieve the reparation of periodontal tissues. Four dental tissues (i.e., gingiva, alveolar bone, cementum, and periodontal ligament [PDL]) make up the periodontium, which relies on the excellent differentiation capacity into cementoblasts, odontoblasts, and fibroblasts of periodontal ligament stem cells (PDLSCs). PDL is an essential constituent part in the attachment of teeth to the jaw, and PDL destruction could result in the loss of the tooth in severe cases of periodontitis commonly caused by a chronic inflammation [106]. Therefore, suitable acellular biomaterials, capable of healing periodontal sites by recruiting autologous cells into PDL scaffolds, should be fabricated and employed to provide in-situ regeneration in cases of periodontitis in the repatriation of periodontal tissues [51]. Compared to SF alone, GO or GO plus fibroin-coated surfaces improved F-actin content, cell spreading and growth, the initial cell adhesion of PDLSCs, and cell activity. In addition, the biomaterials employed proved to be capable of maintaining the mesenchymal phenotype of PDLSCs.

### 3.2. Tissue Engineering

#### 3.2.1. Bone and Cartilage Tissue Engineering

Bone regeneration, rather than autologous or allogeneic bone transplantation, due to limited supply, is required in complex clinical conditions, including skeletal reconstruction of significant bone defects created by trauma, infection, or tumor resection [16,107]. Carbon-based materials, particularly graphene-based biomaterials, have been demonstrated to enhance the osteogenesis ability of mesenchymal stem cells in bone tissue engineering, further offering a viable approach for bone regeneration [108,109,110].

A graded cGO-HA/SF biomimetic scaffold was fabricated Wang et al. by dispersing GO and hydroxyapatite with chitosan solution, blending with SF, and freeze-drying the resulting mixture [111]. The scaffolds obtained were composed of a gradient structure similar to that of a natural bone. The resulting cGO-HA/SF scaffolds could mimic cortical bone, cancellous bone, and the medullary cavity with increased porosity and decreased density from outside to inside. The results showed that the compressive strength of cGO-HA (1:4)/SF was much higher than that of natural cancellous bone (4–12 MPa) and up to 50–60% of that of natural compact bone (130–180 MPa). On account of the scaffolds’ distinct gradient structure and excellent biocompatibility, related assays indicated enhancement of cell attachment and osteogenic differentiation of mouse mesenchymal stem cells (mMSCs). Then, Wang et al. prepared a SF/GO composite scaffold via freeze-drying [112]. The pore size decreased with the addition of GO content, increasing at specific concentrations, without a decrease in porosity. Meanwhile, there was a gradually increasing trend for composites in terms of enhancing osteoprogenitor growth and proliferation with the increased incorporation of GO, indicating the great potential of SF/GO scaffolds in bone tissue repair and regeneration.

An ultrafine nanofiber scaffold was fabricated via the electrospinning method [36]. The composite contained a mixture of TSF, GO, and poly(L-lactic-co-glycolic acid) (PLGA). The incorporation of GO and TSF improved the tensile strength and Young’s modulus of the PLGA matrix. Due to the enhanced extracellular matrix, cell attachment, proliferation, and osteogenic differentiation of mMSCs improved significantly.

Narimani et al. prepared a novel SF/GO nanocomposite scaffold through chemical crosslinking and freeze-drying [113]. The existence of GO nanoparticles enhanced the mechanical properties of the scaffolds but reduced the porosity. The results suggested that the composite could promote cell adhesion and osteogenic differentiation of human osteoblast cells (MG-63).

As the studies presented indicated, the synergistic effects of SF and GO on physicochemical properties and cellular behaviors provide a promising strategy in bone tissue engineering.

The prepared tough photocrosslinked RSF/GO nanocomposite hydrogels, equipped with characteristics such as superb tensile mechanical properties, an increased β-sheet content, and decreased chain mobility, represent promising biomaterials. The will be usefully employed in load tissue engineering thanks to their higher tensile mechanical properties than those of certain natural elastomers (cork, cartilage, and skin) in both dehydrated and swollen states [20].

However, a SF/GO substrate without a 3D structure may limit osteogenesis and angiogenesis compared to a 3D scaffold. Therefore, the in-vivo effects of an SF/GO composite within a 3D environment should be studied further. A novel 3D porous SF/GO nanocomposite scaffold was prepared through freeze-drying technology [113]. Apart from the increase in the mechanical properties and cellular responses of human osteoblast cells (MG-63), the water uptake of the scaffold was also enhanced. The total porosity was decreased, possibly due to the incorporation of GO [113,114]. This showed that a more significant content of SF was likely to reach a higher porosity. Still, the smaller pore size of the SF/GO composite scaffolds was not harmful to the biological behavior compared to that of an SF scaffold [113]. An increase in toughness with the introduction of GO or reduced GO (rGO) into carboxymethyl cellulose (CMC)/SF films [115]. The hydrophilicity, swelling degree, and degradability were demonstrated to present a decreasing trend with the SF content increasing, whereas the tensile strength increased. An increase in the rGO concentration led to a decrease in the porosity and tensile strength and an improvement of the hydrophilicity. The CS content largely contributed to enhancing the cell behaviors of G-292 cells.

Moreover, injectable SF/GO hydrogel scaffolds have been fabricated and characterized as an excellent biomedical material in bone tissue engineering [116]. The effects of introducing other biomaterials into an SF/GO composite have been widely investigated. Increasing the CS content could facilitate the attachment of G-292 cells [114]. GO can act as a slow-release carrier due to its π–π stacking, hydrogen bonding, and electrostatic interactions between proteins [80]. Therefore, Wu et al. combined P24, a new 24-amino acid polypeptide originating from the knuckle epitope of BMP-2, with similar osteogenic activity to BMP-2, with GO to overcome the short half-life of BMP-2 [80].

However, few studies have applied the composites of SF and graphene-family materials to cartilage tissue regeneration.

#### 3.2.2. Neural Tissue Engineering

Neuroregeneration is a therapeutic process of repairing the injured neurons resulting from neurodegenerative diseases. The primary condition of neural restoration is to provide nerve tissues with electrical activity, and graphene-based materials can meet this requirement [117]. To some degree, SF possesses mechanical properties similar to natural nerve tissues [118]. Therefore, the combined properties of SF and graphene-family biomaterials present much potential in applying neural tissue engineering.

Electrospun SF scaffolds coated with rGO were produced as an electroactive conductor to stimulate or restore the functionality of damaged neural tissues [119]. When cultured with rat adrenal phaeochromocytoma PC-12 cells, the composites exhibited excellent biocompatibility and improved the cell attachment compared to cells grown on the pure SF scaffolds. Remarkably, the application of SF/rGO scaffolds combined with electrical stimulation played a crucial role in promoting the differentiation of PC-12 cells into neural phenotypes without relying on a neural growth factor. Zhao et al. fabricated a composite nanofiber membrane of SF and graphene via electrospinning, mimicking a natural neural cell micro-environment for nerve development by graphene’s magnetic electrical and mechanical properties and the biocompatibility of silk [120]. In another study, the SF/graphene conductive film enhanced induced pluripotent stem cells’ (iPSCs’) differentiation into neurons, with the graphene contents increasing, reaching the highest value at a 4% content [121]. However, a high content of graphene (over 8%) inhibited cell differentiation.

### 3.3. Biosensor

Biosensors, applied for monitoring biological processes and detecting biomolecules, are expected to meet some requirements, including having excellent physicochemical properties, biocompatibility, conductivity, and biostability [122]. Graphene presents excellently high sensitivity relying in terms of its crystal lattice [123]. The combination of SF and graphene-based materials has attracted much attention due to the integration of high flexibility and electrical conductivity. Considering the double advantages of the extremely high sensitivity of the graphene work and the transferability of the water-soluble SF platform, a kind of graphene-SF nanosensor was established to apply wireless bacteria detection to tooth enamel [124]. It was concluded that the G-SF composite biosensors supported some novel strategies for developing electrically conductive biomaterials, bendable electrodes, and wearable biomedical devices [125]. Recently, an electronic smell biosensor, based on the *Bombyx mori* pheromone binding protein (BmorPBP1) immobilizing on an rGO surface of a field-effect transistor, was developed to detect odors, among which only eugenol could elicit a strong signal [126].

### 3.4. Drug Delivery

It has been concluded that the release of drugs from scaffolds is governed by several elements, such as the nature and molecular weight of the drug, degree of crosslinking density, the pore size of the matrix, solvent type, and so on [127]. The porous SF/GO composite scaffolds presented a drug release rate that decreased with the increase in the GO content when adopting simvastatin (SIM) as the drug model, which is beneficial for the growth and proliferation of osteoblasts [23]. Delaying the drug release relied mainly on two factors [23]: ① the existence of hydrophobic-hydrophobic interactions between a silk II structure and hydrophobic SIM molecules led to an increasing drug residence time in the scaffolds; ② the GO nanosheets could act as physical crosslinking points depending on the formation of hydrogen bonds between the oxygen-containing groups of GO and the polar side-chain groups in the SF molecule. A sustained drug-release effect is advantageous to better match drug delivery and bone tissue regeneration.

Salvianolic acid (SB), a traditional Chinese herbal medicine with various biological activities, has been reported to promote spinal fusion by facilitating osteogenesis and angiogenesis [128]. An SF-GO composite scaffold loaded with large doses of SB was synthesized and achieved the synergistic effect of SB and GO in osteogenesis and angiogenesis through both physical adsorption and self-assembly between hydroxyl groups of SB carboxyl groups of GO [129]. With an effective loading rate of 44.14%, the scaffolds could maintain the biological activity of SB during the continuously releasing process.

Robust (SF-PL/GO)_n_ composite microcapsules showed excellent mechanical properties with an elastic modulus value of about 0.5 GPa, a capacity to maintain stability under extreme acid (pH 2) and primary (pH 11.5) conditions, and pH-regulated permeability [22], thereby representing a promising biomaterial for drug delivery, cell surface engineering, and biosensors under harsh environmental conditions [130]. Beyond this, there is much need and great feasibility for these composite microcapsules to be investigated in terms of other properties such as cytocompatibility, degradability, and hydrophilicity via in-vitro and in-vivo tests. A brief description of the applications of the graphene-based scaffolds in different tissue engineering types is provided in Table 1. Similarly, commercial, preclinical, and clinical-stage materials description is provided in Table 2.

## 4. Conclusions

Silk fibroin, a natural polymer material, has been widely applied in the biomedical fields mainly owing to its advantages, such as proper biocompatibility, adaptable biodegradability, stable mechanical properties, and the ability to be modified easily. There are three types of materials in the graphene family—graphene, graphene oxide, and reduced graphene oxide—possessing excellent mechanical properties, electrical conductivity, and atomic structural stability. The combination of silk fibroin and graphene-based materials offers the potential to mix their advantages and cover each other’s shortcomings, to produce enhanced functions with more significant potential in biomedical fields. However, biotoxicity and biodegradability remain challenges that need further study. Meanwhile, there is still a need to further investigate the specific signal pathway of the mechanisms of the effects of such hybrid composites on cells. Various fabrication methods have been reported for composite biomaterials, such as freeze-drying, layer-by-layer assembly, electrochemical microstamping, crosslinking, etc. Recently, the combinations of silk fibroin and graphene family materials have shown great popularity in the biomedical fields, including tissue engineering, drug delivery, and dental fields. In oral and maxillofacial surgery, tissue engineering (bone, nerve, cartilage) has been a research topic in great demand. More efforts should be made to identify new information that supports new treatments for common oral and maxillofacial issues such as maxillofacial fractures and tumors.

## Figures and Tables

**Table 1:** Application of graphene-based composite scaffolds in different tissue engineering types.

Tissue type	Origin	Composition of the Scaffold	Method	Characteristics	Ref.
Bone tissue engineering	Graphene-based natural biomaterial	Gelatin/Hydroxyapatite/GO	Solvent casting	GO supporting cell adhesion, increasing cell activity and alkaline phosphatase secretion, inducing bone regeneration	[131]
		Silicate/Hydroxyapatite/GO	Electrospinning	Better adhesion, spreadability, proliferation, and alkaline phosphatase activity	[81]
		Gelatin/Sodium alginate/GO	Freeze-drying	Improved hydrophilicity and mechanical properties, prolonged biodegradation time, inducing bone regeneration	[82]
		Phosphate/Graphene	Soft template	Hierarchical porous structure, accelerating bone repair	[132]
		Poly(β-caprolactone)/Graphene	Arbuzov reaction	Inducing bone regeneration driven by stem cells	[133]
		GO/PLA	3D printing	Low immunogenicity, which enhances the adhesion and reactivity of growing cells and promotes bone formation	[134,135]
		GO/PCL/CHT/Collagen	Melt deposition molding	Young’s modulus increasing by 30%, good biocompatibility, and promoting cell proliferation and bone mineralization	[136]
	Graphene-based natural synthetic biomaterials	Silk/GO	Electrospinning	Good morphology and biocompatibility, which can improve the cell activity to complete damage repair	[137]
Nerve tissue engineering	Graphene-based natural biomaterial	CHT/GO	Electrospinning	Enhancing the metabolic and proliferative activities of nerve cells	[138]
	Graphene-based natural synthetic biomaterials	PCL/Gelatin/Graphene	Electrospinning	Good biocompatibility, which enhances the adhesion and differentiation of nerve cells	[139]
		GO/CHT		The scaffold has good conductivity and cell compatibility, which can provide a suitable microenvironment for nerve tissue regeneration	[140]
Cardiovascular tissue engineering	Graphene-based natural biomaterial	Collagen/rGO	Freeze-drying	Enhancing the expression of specific myocardial proteins and genes and promoting the adhesion and aggregation of myocardial cells	[141,142]
	Graphene-based synthetic biomaterials	Graphene/PCL	Electrospinning	Good biocompatibility, antipressure ability, which meets the requirements of normal blood vessels, low platelet adhesion, and activation ability	[143]
		Gelatin/PCL/Paramagnetic iron oxide/GO	Chemical vapor precipitation	Enhancing the structural characteristics of myocardial cells, and increasing the expression of cell–cell coupled protein and calcium-treated protein	[144]
	Graphene-based natural synthetic biomaterials	RGD peptide/GO/Poly (lactide/glycolide)	Blending method	Good histocompatibility and blood compatibility, being effectively monitored by magnetic resonance imaging	[145]
Skin tissue engineering	Graphene-based natural biomaterial		Freeze-drying	Providing a good microenvironment for the growth and proliferation of stem cells and contributing to the healing of diabetic wounds	[146]
	Graphene-based synthetic biomaterials	PCL/PU/GO	Electrospinning	Improving the hydrophilicity, biocompatibility, and stability of the scaffold by GO, stimulating the proliferation of human skin fibroblasts	[147]
Muscle tissue engineering	Graphene-based synthetic biomaterials	PU foam/Graphene oxide	Electrospinning	Inducing the formation of multinucleated myotubes, promoting the adhesion and proliferation of C2C12 mouse myoblasts	[148]

**Table 2:** Commercial-, preclinical-, and clinical-stage materials.

Purpose	Commercial	Pre-Clinical	Clinical	Reference
Wound healing		Yes		[149]
Wound healing		Yes	Yes	[150]
Biomedical		Yes		[151]
Drug delivery		Yes		[152]
Hydrogels	Yes			[6]

## Data Availability

Not applicable.
